# Supplementary material for: Altered ubiquitin signaling induces Alzheimer’s disease-like hallmarks in a three-dimensional human neural cell culture model
Source: Nat Commun. 2023 Sep 22;14:5922. doi: 10.1038/s41467-023-41545-7 (PMC10516951; doi:10.1038/s41467-023-41545-7)

Supplementary Information

Altered ubiquitin signalling induces Alzheimer's disease-like hallmarks in a three-dimensional human neural cell culture model

Inbal Maniv[1,*], Mahasen Sarji[1,*], Anwar Bdarneh[1,*], Alona Feldman[1], Roi Ankawa[1], Elle Koren[1], Inbar Magid-Gold[1], Noa Reis[1], Despina Soteriou[1], Shiran Salomon-Zimri[2], Tali Lavy[1], Ellina Kesselman[3], Naama Koifman[3], Thimo Kurz[4], Oded Kleifeld[1], Daniel Michaelson[2], Fred W. van Leeuwen[5], Bert M. Verheijen[1,5], Yaron Fuchs[1,&,#] and Michael H. Glickman[1,#]

[1] Department of Biology, Technion Israel Institute of Technology, Haifa Israel 32000003.
[2] Department of Neurobiology, The George S. Wise Faculty of Life Sciences, The Sagol School of Neuroscience, Tel Aviv University, Tel Aviv 69978, Israel.
[3] The Wolfson Department of Chemical Engineering, The Technion Center for Electron Microscopy of Soft Matter, Technion Israel Institute of Technology, Haifa, 3200003, Israel.
[4] School of Molecular Biosciences, University of Glasgow, Glasgow G12 8QQ Scotland UK
[5] Department of Neuroscience, Maastricht University, 6229 ER Maastricht, the Netherlands

[*] These authors contributed equally
[&] Current address Augmanity, Rehovot 7670308 Israel.
[#] Correspondence to: yaronfox@gmail.com and glickman@technion.ac.il.

This file contains :

1 Supplementary Table p.2
5 Supplementary Figures (1-5) p. 3-12
Source data files (for supplementary figures) p. 13-

Case	Age (years)	Sex	Neuropathological diagnosis	Braak stage	Disease duration (years)[a]	PMI (hours)	Fixation duration (days)	Brain weight (g)	Cause of death
1	58	M	Ctrl.	0	-	24	1088	1797	Lung carcinoma, massive hemorrhage
2	65	F	Ctrl.	0	-	24	403	1234	Pulmonary embolism
3	82	F	Ctrl.	I	-	48	38	1100	Myocardial infarction, ventricular fibrillation
4	90	F	Ctrl.	I	-	5	143	1040	Metabolic acidosis
5	67	F	Ctrl.	I	-	70	NA	1158	Diabetes, decompensatio cordis
6	72	M	Ctrl.	II	-	4	126	1330	Myocardial infarction, cardiogenic shock
7	80	F	Ctrl.	II	-	36	65	1205	Cardiogenic shock
8	85	M	Ctrl.	III	-	5	126	1050	Cardiac failure, myocardial infarction, coronary sclerosis, lung emphysema
9	77	M	AD	II	>5	4	127	1095	AD, Bronchial pneumonia
10	88	M	AD	III	4	5	75	1058	AD, decompensatio cordis
11	86	M	AD	V	10	4	77	1303	AD, uraemia
12	85	F	AD	V	NA	2.5	NA	1020	AD
13	66	M	AD	VI	15	3	30	1270	AD, ischemic cerebral stroke, cachexia, sepsis
14	70	F	AD	VI	12	13	34	780	AD, status epilepticus

Supplementary Table 1. Cases included in the present study.

Supplementary Table 1. Cases included in the present study. Dentate gyrus (DG) of patients diagnosed at different Braak stages. Abbreviations: Alzheimer's disease (AD), Control (Ctrl.), Female (F), Male (M), Not available (NA), Post-mortem interval (PMI).

[a] Disease duration refers to the duration of dementia.

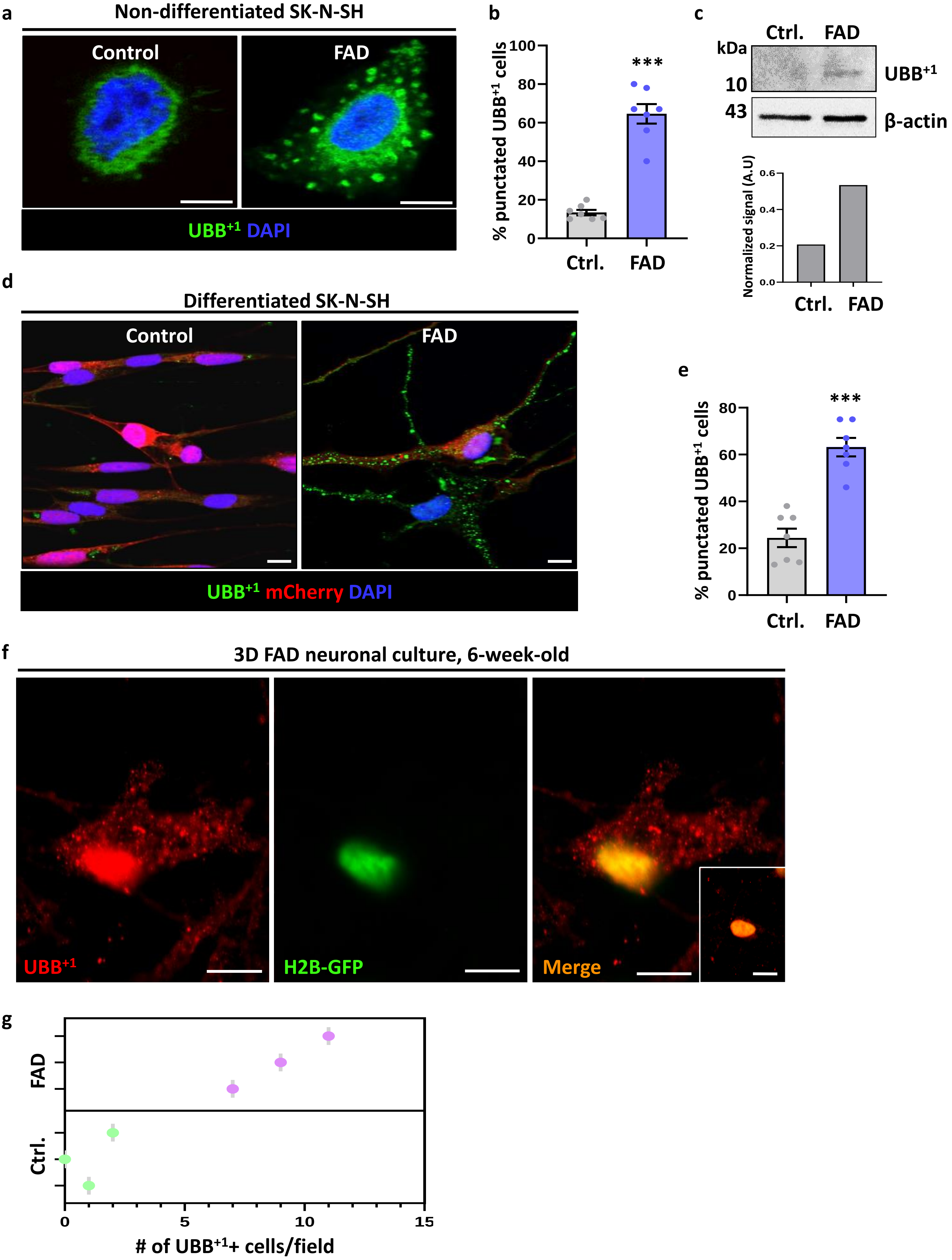

Supplementary Figure 1. UBB^{+1} accumulates in different AD models.

Supplementary Figure 1. UBB^{+1} accumulates in different AD models. a Immunofluorescence of control and FAD-expressing SK-N-SH cells stained with anti-UBB^{+1}. **b** Quantification of puncta in UBB^{+1}-positive cells from (**a**) [n=7 biologically independent samples] (p=0.0000006). **c** Anti-UBB^{+1} immunoblot of extracts from control and FAD-expressing SK-N-SH cells (top panel). Densitometry of UBB^{+1} signal normalized to β-actin (bottom panel). **d** Immunofluorescence with UBB^{+1} antibody of three-week-old differentiated SK-N-SH cells. **e** Quantification of UBB^{+1} puncta in differentiated cells (from **d**). [n=7 biologically independent samples] (p=0.000016). **f** Representative images of six-week-old FAD ReNcell 3D cultures stained for UBB^{+1}. Inset shows a control culture stained for UBB^{+1}. **g** Quantification of UBB^{+1} positive cells/field (field=25 cells) from **f** [n=3 biologically independent samples]. *P* values were determined by unpaired two-tailed Student's *t*-test. Error bars represent ± s.d. Images are representative of three independent wells. All experiments were repeated at least twice. Scale bars: 10 µm (**a, d**) 20 µm (**f**).

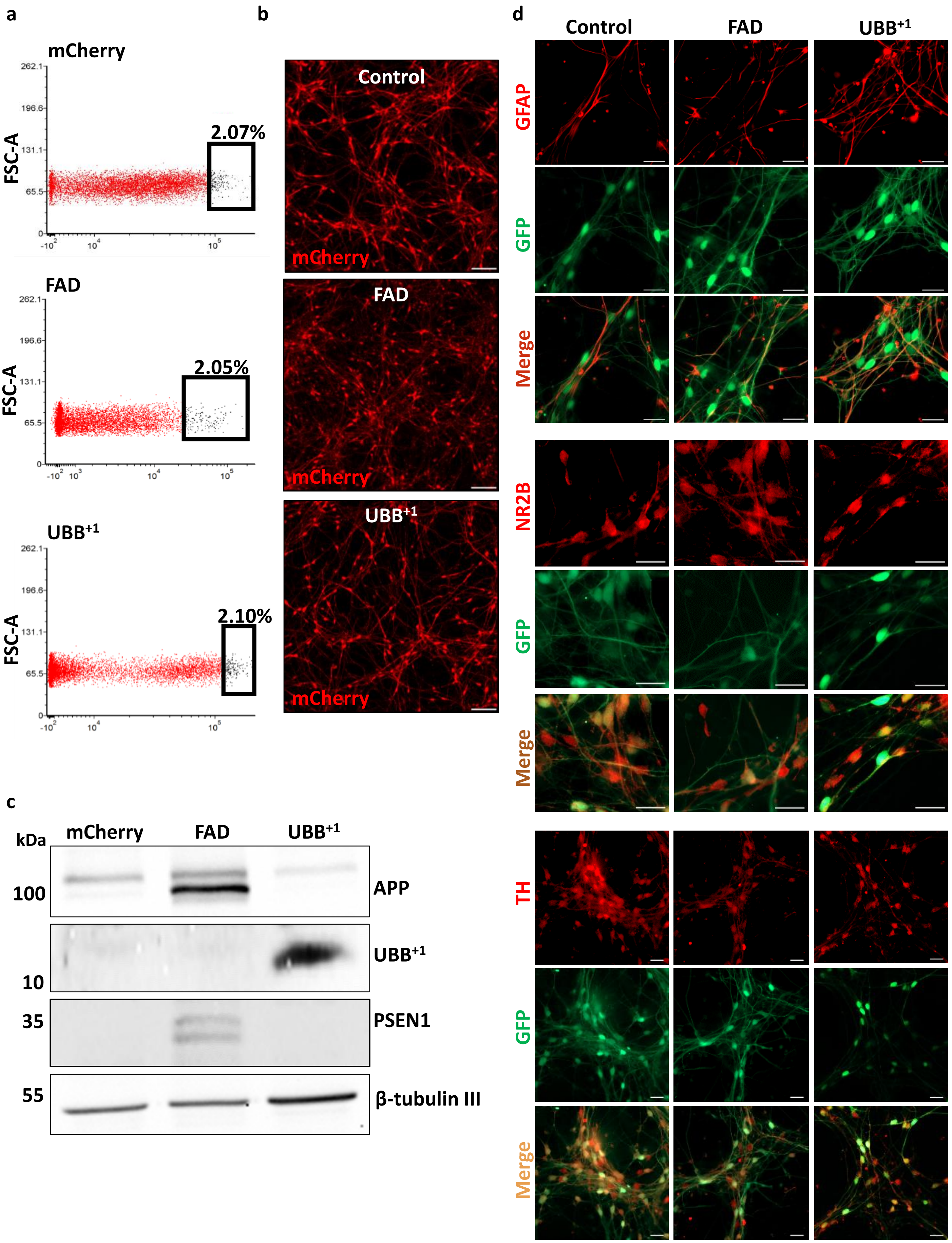

Supplementary Figure 2. Establishment of a 3D human neuronal AD model.

Supplementary Figure 2. Establishment of a 3D human neuronal AD model. a Sorting of ReN cells that were transduced with polycistronic mCherry lentiviral vectors: Control (mCherry), FAD (APP^{SL}-$PSEN1^{\Delta E9}$–mCherry), and UBB^{+1} (UBB^{+1}-mCherry). The cells were enriched based on the mCherry signal. Black-dotted boxes indicate the sorted cell population (top 2%). **b** Representative fluorescence confocal microscopy images showing expression of mCherry in ReN cells differentiated by growth-factor deprivation for three weeks. **c** Western blot analysis of APP^{SL}, PSEN1ΔE9, and UBB^{+1} ReN cells, showing expression of proteins following transduction. **d** Immunostaining for GFAP, NR2B, and TH expression indicates proper differentiation of six-week-old neuronal cultures. Images are representative of three independent wells. All experiments were repeated at least twice. Scale bars: 50 µm (**b**), 20 µm (**d**).

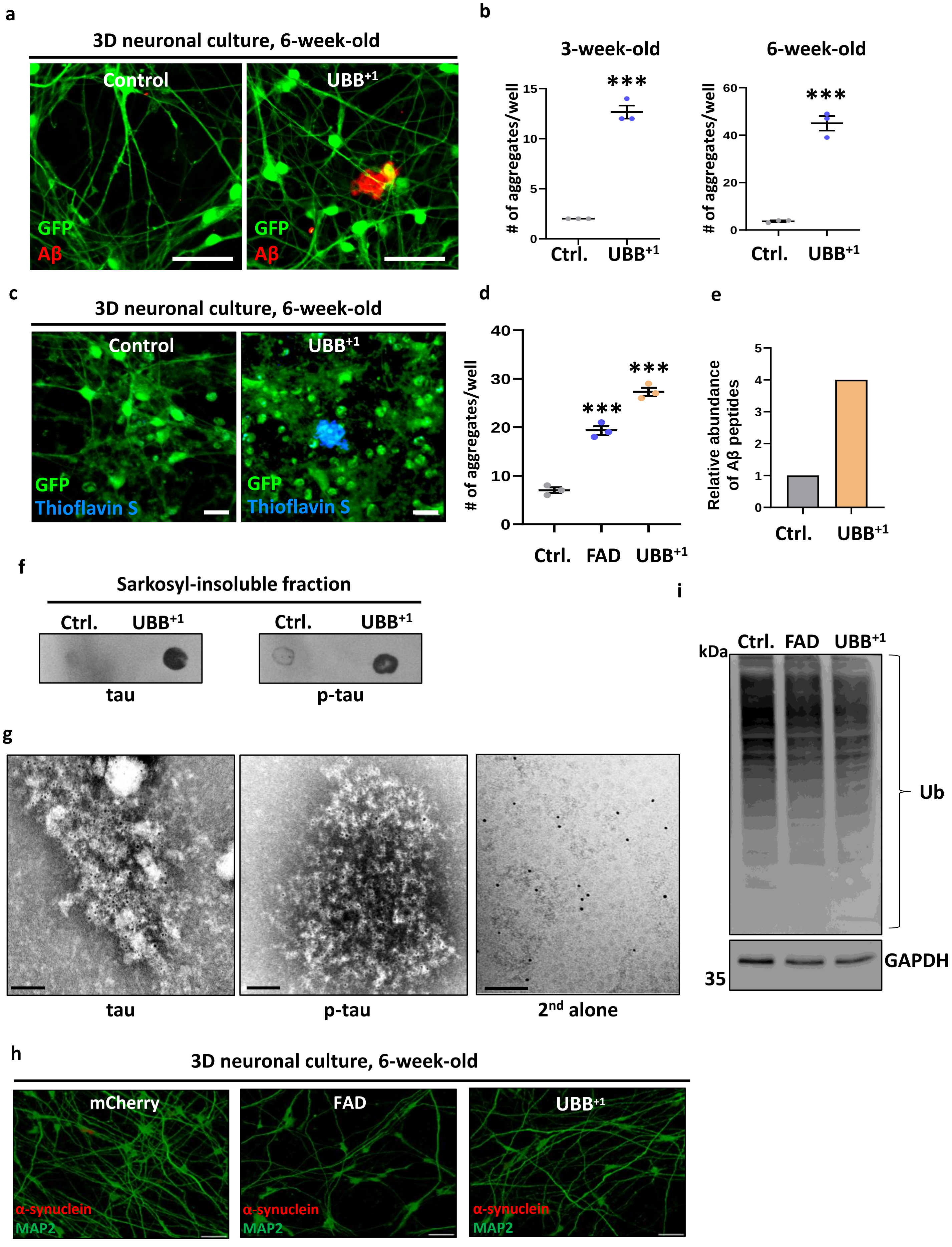

Supplementary Figure 3. Expression of UBB+1 is sufficient to induce Aβ42 and tau-positive aggregates in a 3D human neuronal culture.

Supplementary Figure 3. **Expression of UBB^{+1} is sufficient to induce Aβ42 and tau-positive aggregates in a 3D human neuronal culture.** **a** Representative images of six-week-old control and $UBB^{+1(D79S)}$ (UBB^{+1} with a D79S amino acid substitution) cultures stained with a specific anti-Aβ antibody (3D6, in red), showing staining of aggregates in between cells. **b** Quantification of the total number of $Aβ^{+}$ aggregates (3D6) in three- and six-week-old control and $UBB^{+1(D79S)}$ 3D cultures [n=3 biologically independent samples] (three-week-old p=0.00009; six-week-old p=0.0002). **c** Representative images with Thioflavin S staining of six-week-old control and $UBB^{+1(D79S)}$ cultures, showing aggregates in between cells. **d** Quantification of the total number of Thioflavin S^{+} aggregates in six-week-old control, FAD, and $UBB^{+1(D79S)}$ 3D cultures [n=3 biologically independent samples] (Ctrl./FAD p= 0.0003; Ctrl./UBB^{+1} p=0.00004) **e** SDS-soluble fractions extracted from six-week-old $UBB^{+1(D79S)}$ and control cultures, showing a four-fold increase in Aβ peptide by MS/MS quantification. A comparison was done by calculating the peak area of each peptide [n=1]. **f** Dot-blot analysis using T46 and PHF1 antibodies of sarkosyl-insoluble fractions extracted from eight-week-old 3D UBB^{+1} and control cultures. **g** Representative negative stained immuno-TEM of sarkosyl-insoluble fractions from twelve-week-old UBB^{+1} 3D cultures using T46 (left) or PHF1 (right) antibodies followed by 5 nm nano-gold particles conjugated secondary antibodies. **h** Representative immunofluorescence images using anti-alpha-synuclein of six-week-old control, FAD, and UBB^{+1} 3D cultures. **i** Immunoblot of total ubiquitin levels from eight-week-old 3D control, FAD and UBB^{+1} cultures, showing no difference in total ubiquitin levels between all three cultures. *P* values were determined by unpaired two-tailed Student's *t*-test. Error bars represent ± s.d. Images are representative of three independent wells. All experiments were repeated at least twice. Scale bars: 50 µm (**a**), 20 µm (**c**), and 100 nm (**g**), 50 µm (**h**).

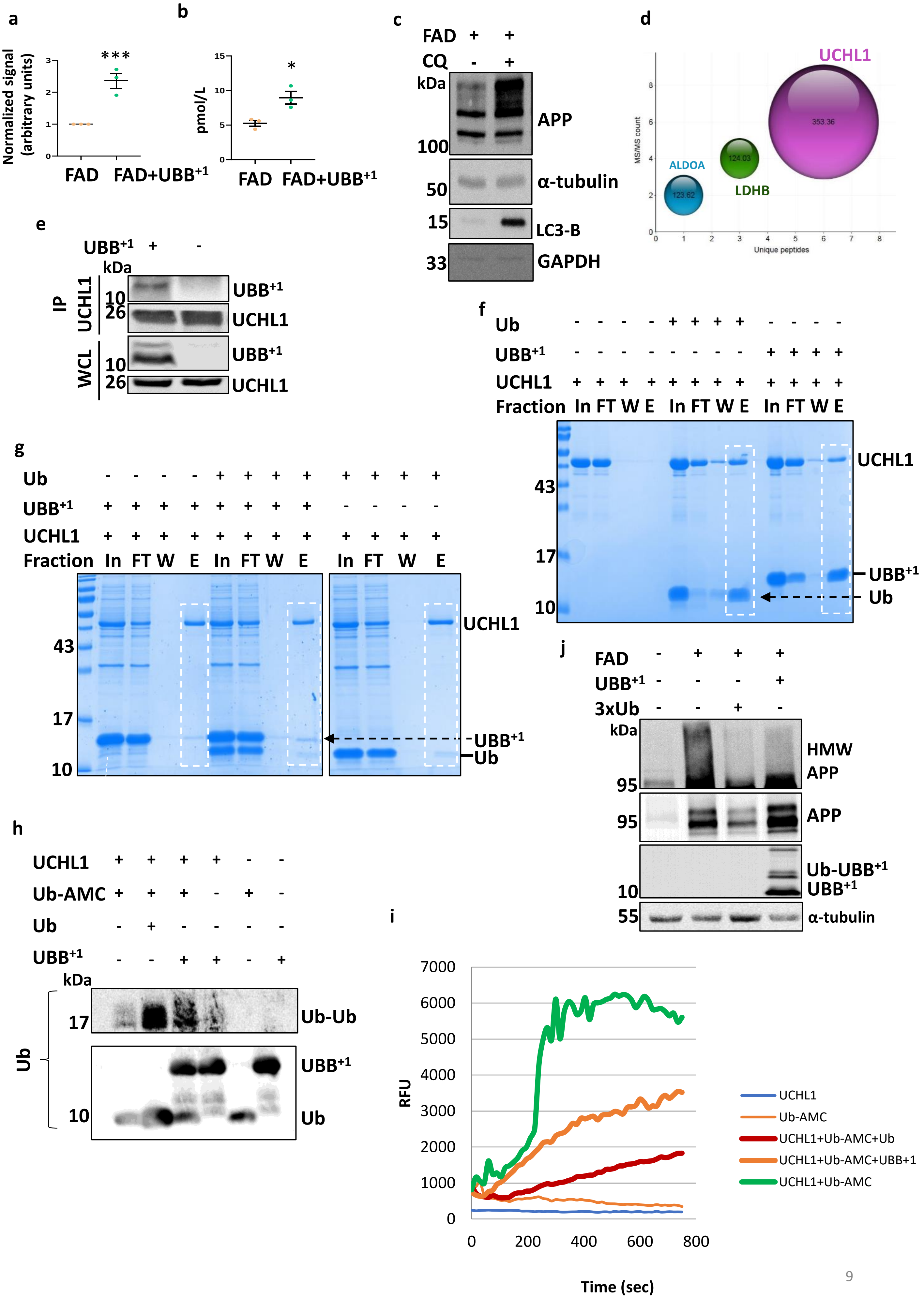

Supplementary Figure 4. UBB+1 binds UCHL1 and interferes with its catalytic activities.

Supplementary Figure 4. UBB^{+1} binds UCHL1 and interferes with its catalytic activities. a Quantification of Aβ in conditioned media (as in Fig. 3a) by densitometry of immunoblots. The signal was normalized to the signal of tubulin in whole cell lysate WCL. [n=3 biologically independent samples]. **b** Aβ42 protein levels quantified by ELISA from conditioned media of HEK293FT cells expressing FAD or FAD +UBB^{+1}. [n=3 biologically independent samples] (p=0.02). **c** Immunoblot of APP in HEK293FT cells transfected with FAD and treated with Chloroquine lysosome inhibitor, which prevents endosomal acidification. APP levels are increased by lysosome inhibition. GAPDH was used as loading control and LC3-B verified lysosome inhibition. **d** Mass spectrometry (MS/MS) analysis of proteins that co-purified with immunoprecipitated UBB^{+1} in HEK293FT cells. The most abundant candidates are presented according to the number of unique peptides (x-axis), the total MS/MS count (y-axis), and the total MS peak intensity (bubble size). **e** Co-IP of expressed UBB^{+1} in HEK293FT cells with endogenous immunoprecipitated UCHL1 using anti UCHL1 specific antibody. **f** *In vitro* binding assay. Immobilized His-ubiquitin or His-UBB^{+1} were incubated with recombinant GST-UCHL1. Following elution with imidazole, proteins were resolved by SDS-PAGE and stained with Coomassie. Input (In), flow-through (FT), wash (W), elution (E). **g** *In vitro* binding assay. Immobilized GST-UCHL1 was incubated with recombinant His-ubiquitin or His-UBB^{+1}. Following elution with GSH, proteins were resolved by SDS-PAGE and stained with Coomassie. Input (In), flow-through (FT), wash (W), elution (E). **h** *In vitro* ligase assay. UCHL1 was incubated with ubiquitin-AMC for three hours, reaction products were resolved by SDS-PAGE and stained for ubiquitin. Ubiquitin dimers were increased upon the addition of 17xfold unmodified ubiquitin (second lane from the left), but not in the presence of similar addition of UBB^{+1}. **i** Ubiquitin-AMC hydrolysis by UCHL1. UCHL1 was incubated with ubiquitin-AMC alone (in green), or with ubiquitin-AMC and UBB^{+1} (in orange) or ubiquitin (in red). Consider showing only up to 600-700 seconds. Data is shown as Relative Fluorescence Units (RFU) of liberated AMC over time (seconds). **j** HEK293FT cells expressing FAD w/wo UBB^{+1} or 3xUb were immunoblotted for APP. The high molecular weight (HMW) region was overexposed to show modifications (top panel). α-tubulin was used as a loading control. *P* values were determined by unpaired two-tailed Student's *t*-test. Error bars represent ± s.d. All experiments were repeated at least twice, except 4d and 4i.

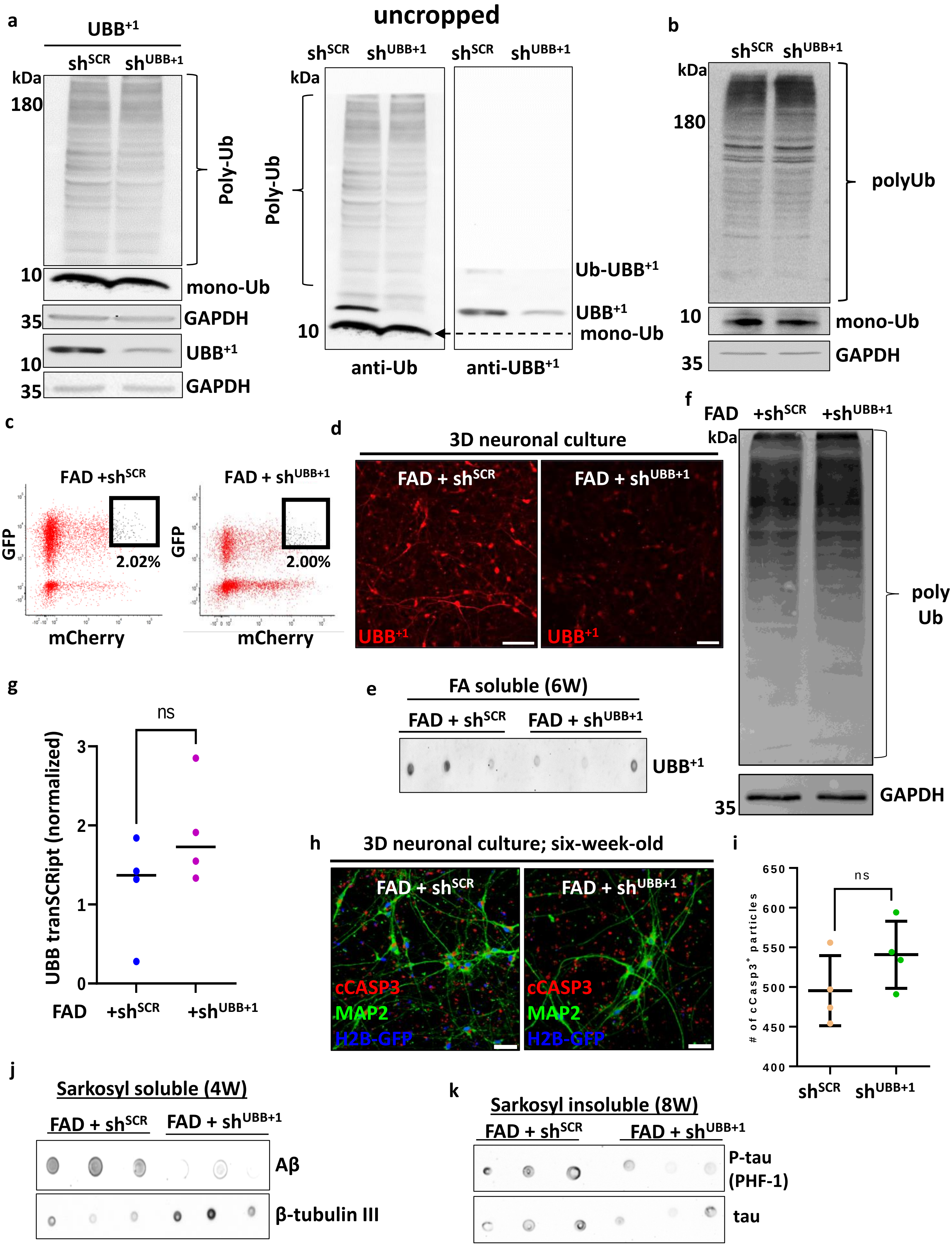

Supplementary Figure 5. Establishment of shRNA for UBB^{+1} in 3D cultures.

Supplementary Figure 5. Establishment of shRNA for UBB^{+1} in 3D cultures. a Immunoblot of HEK293FT cells transfected with UBB^{+1} together with sh^{UBB+1} or sh^{SCR}. sh$^{UBB +1}$ specifically targeted UBB $^{+1}$ (right panel) without affecting other ubiquitin species or conjugates detected in whole cell extract (left panel). **b** Immunoblot of total ubiquitin in HEK293FT cells carrying sh^{UBB+1} or scrambled control. Both HMW and mono-ubiquitin were tested showing that sh^{UBB+1} does not affect total ubiquitin levels in the cell. **c** FACS plots of ReNcell VM that were transduced with polycistronic H2B-GFP and mCherry lentiviral vectors: FAD +sh^{SCR} (APP^{SL}-PSEN1$^{\Delta E9}$–mCherry+SCRambled-H2B-GFP) and FAD+sh^{UBB+1} (APP^{SL}-PSEN1$^{\Delta E9}$–mCherry+shUBB^{+1}-H2B-GFP). Cells were enriched based on GFP and mCherry signals. Black-dotted boxes indicate the sorted cell population (top 2%). **d** Immunofluorescence of 3D neurons expressing sh^{UBB+1} showing a decrease in UBB^{+1} protein levels compared to SCRambled control. **e** Dot-blot analysis of formic acid (FA)-soluble fraction extracted from six-week-old FAD+sh^{SCR} or FAD+sh^{UBB+1} cultures blotted against UBB^{+1} showing decreased levels of UBB^{+1}. **f** Immunoblot of total Ubiquitin levels from eight-week-old 3D FAD+sh^{SCR} or FAD+sh^{UBB+1} cultures, showing no difference in total ubiquitin protein levels. **g** RT-qPCR of RNA extracted from eight-week-old 3D FAD+sh^{SCR} or FAD+sh^{UBB+1} cultures, showing no difference in UBB mRNA levels (normalized to GAPDH). **h** Immunofluorescence of cleaved caspase-3 (cCas3) in six-week-old 3D neurons, showing there is no significant increase of cleaved caspase-3 in accordance to UBB^{+1} overexpression. **i** Quantification of cleaved caspase-3 positive particles in six-weeks-old cultures [n=4 biologically independent samples]. **j** Dot-blot analysis of Sarkosyl-soluble fractions extracted from four-week-old FAD+sh^{SCR} or FAD+sh^{UBB+1} cultures blotted against amyloid beta and beta-tubulin. **k** Dot-blot analysis of Sarkosyl-insoluble fractions extracted from eight-week-old 3D cultures stained for p-tau (PHF1) or tau (MC1). *P* values were determined by unpaired two-tailed Student's *t*-test. Error bars represent ± s.d. Images are representative of three independent wells. All experiments were repeated at least twice. Scale bars: 20 µm (**d**) 50 µm (**h**) .

Sup. Fig. 1b

		Avg	Std	SE	P value
Control	10	13.42857143	3.82087	1.444153	5.60118E-07
	20				
	14.3				
	12.5				
	10				
	10.5				
	16.7				
FAD	64.2	64.6	13.58627	5.135126	
	40				
	67				
	56				
	80				
	78				
	67				

Sup. Fig. 1c

anti UBB^{+1}

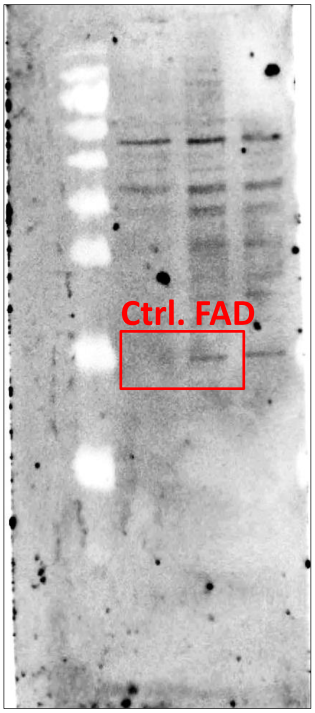

anti actin

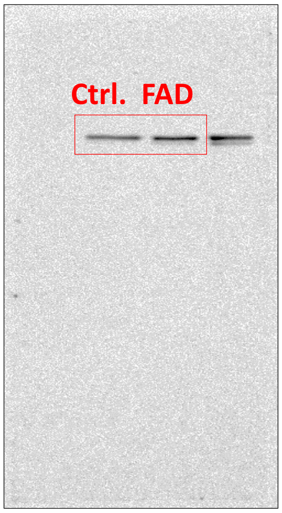

Sup. Fig. 1e

		Avg	Std	SE	P value
**Control**	33	24.42857	10.48582	3.963267	1.58E-05
	15				
	25				
	14				
	38				
	13				
	33				
**FAD**	60	63.14286	10.41519	3.936572	
	46				
	56				
	75				
	75				
	67				
	63				

Sup. Fig. 1g

		Avg	Std	SE	P value
**Control**	1	1	1	0.57735	0.003448
	0				
	2				
**FAD**	7	9	2	1.154701	
	9				
	11				

Sup. Fig. 2c

anti APP (6E10)

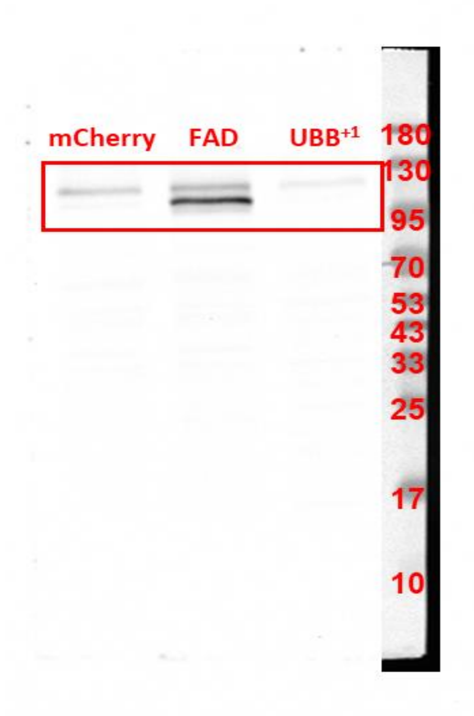

anti PSEN1

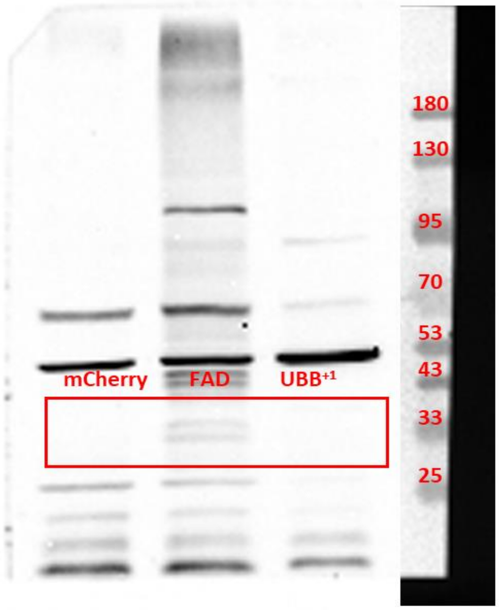

anti β-tubulin III

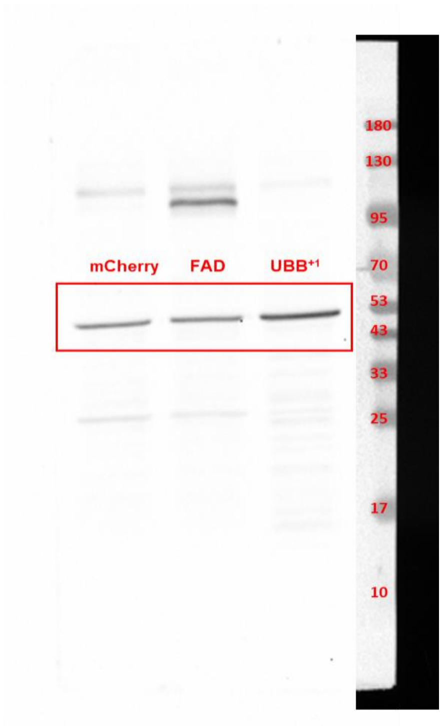

anti UBB^{+1}

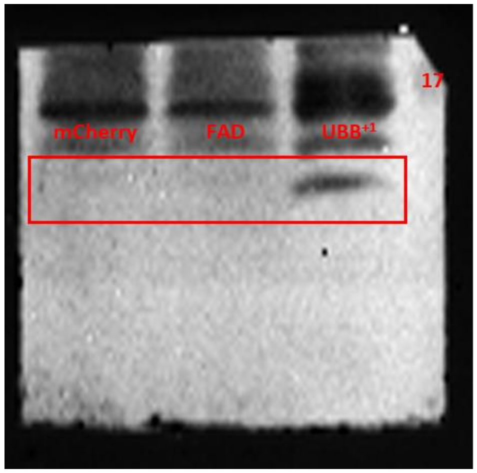

Sup. Fig. 3b-1

		Avg	Std	SE	P value
Control	2				8.92E-05
	2				
	2	2	0	0	
UBB+1	12				
	12				
	14	12.66667	1.154701	0.666667	

Sup. Fig. 3b-2

		Avg	Std	SE	P value
Control	4	3.666667	0.57735	0.333333	0.000177
	4				
	3				
UBB+1	49	45	5.291503	3.05505	
	39				
	47				

Sup. Fig. 3d

		Avg	Std	SE	P value
**Control**	8	7	1	0.57735	0.000305
	7				
	6				
**FAD**	18	19.33333	1.527525	0.881917	
	19				
	21				
**UBB+1**	29	27.33333	1.527525	0.881917	4.26E-05
	26				
	27				

Sup. Fig. 3f

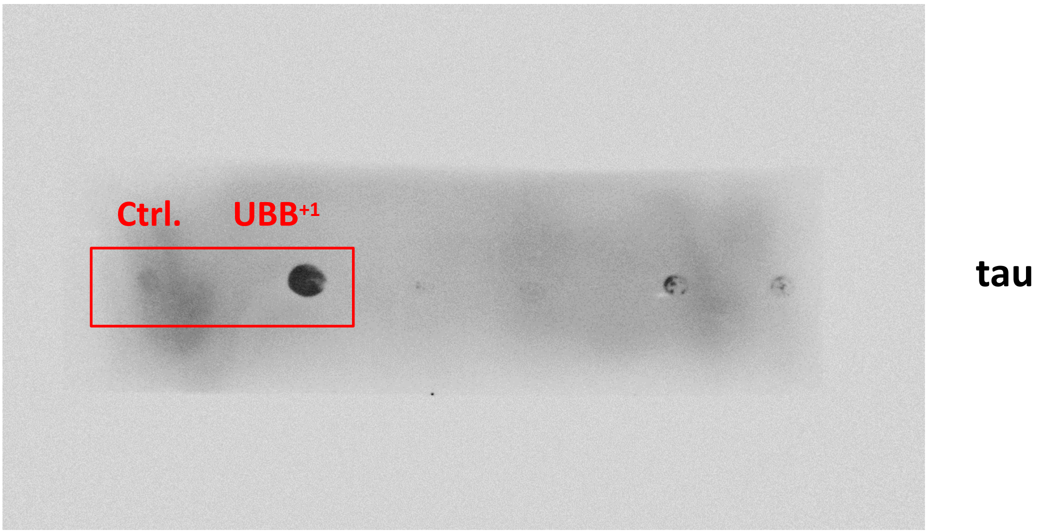

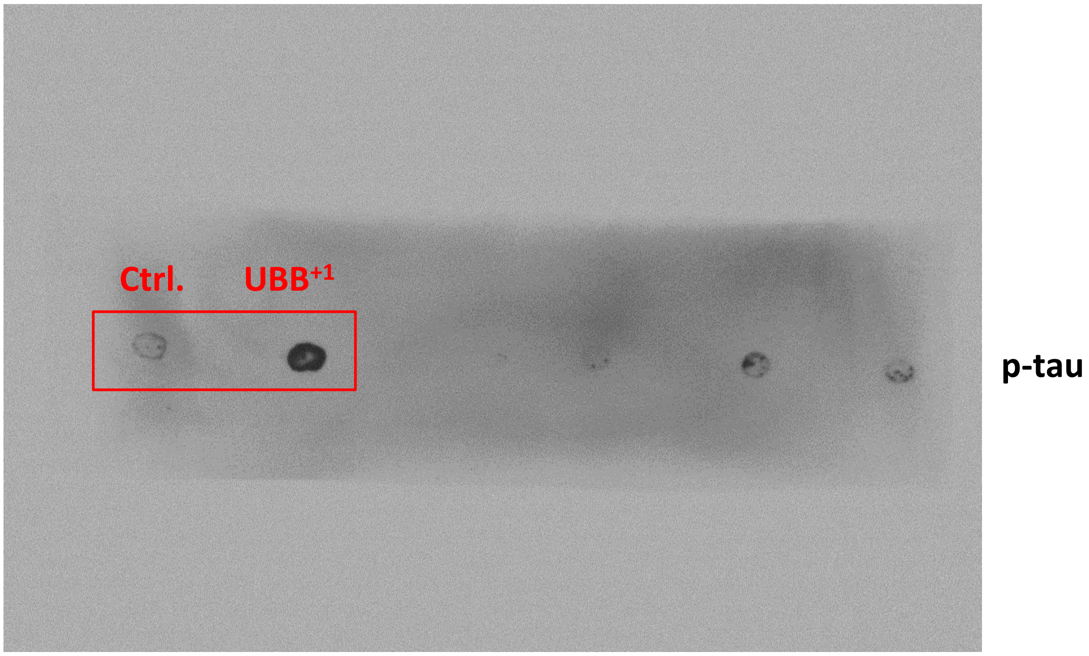

Sup. Fig. 3i

Anti UB

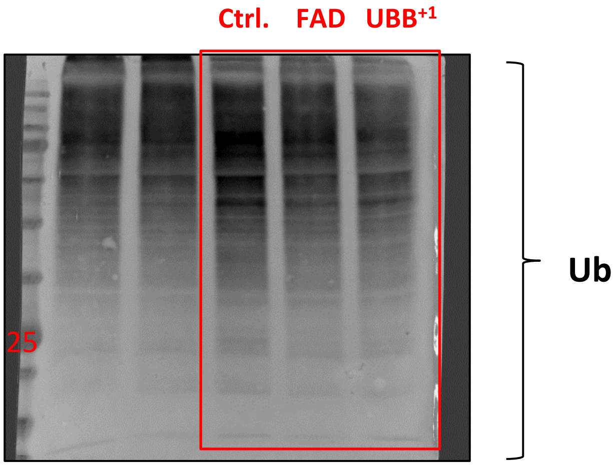

anti GAPDH

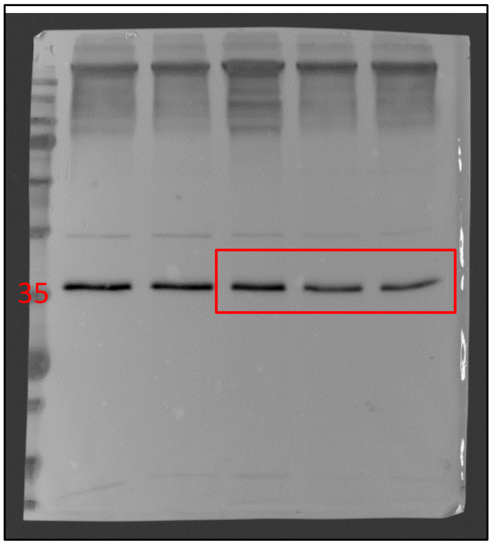

Sup. Fig. 4b

		Avg	Std	SE	P value
**UBB+1**	5.606061				0.022123
	5.833333				
	4.393939	5.277778	0.773816	0.446763	
**UBB+1+FAD**	10.68182				
	8.636364				
	7.575758	8.964647	1.578839	0.911543	

Sup. Fig. 4c

anti APP (6E10)

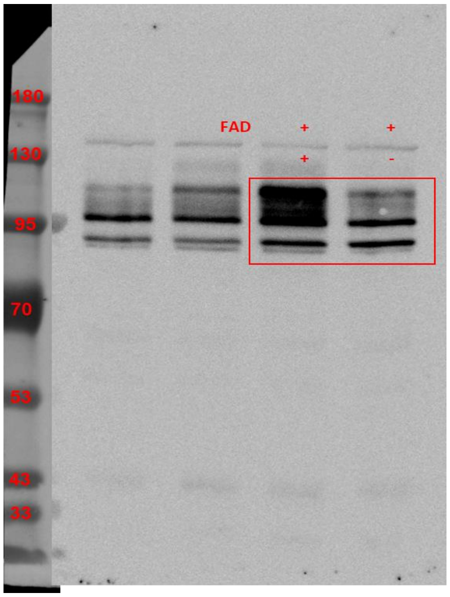

anti LC-3B

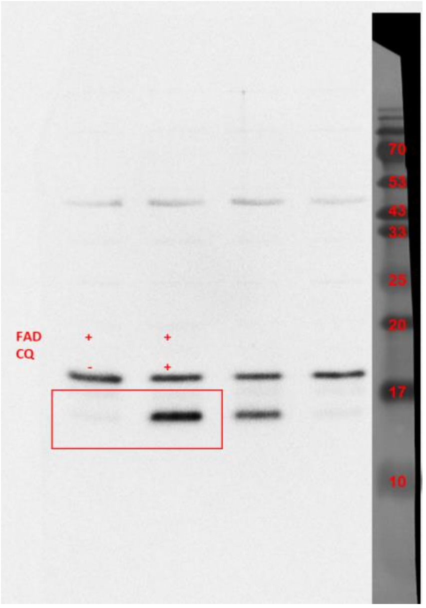

anti tubulin

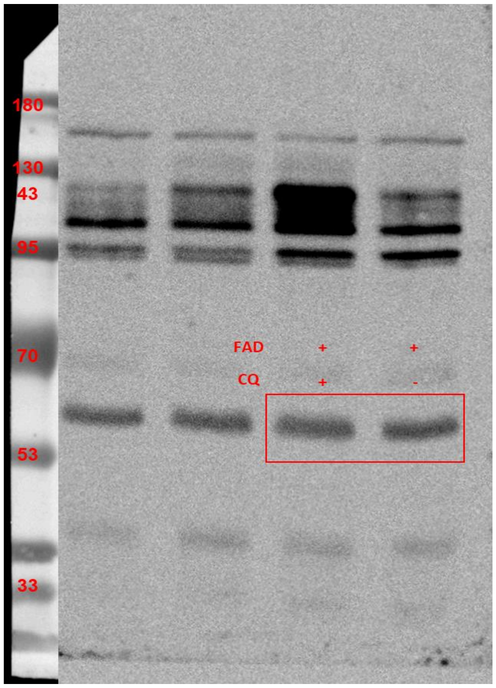

anti GAPDH

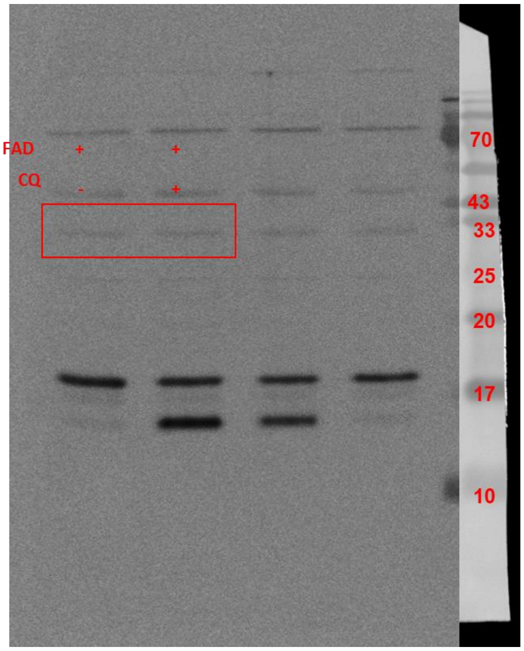

Sup. Fig. 4d

Gene names	MS/MS count IM3	MS/MS count IM4	4_x-y_3	3	4	Unique to 4	4-3>3
**UBB**	**0**	**2**	8.30044	21.3272	29.6277	+	+
**WRNIP1**	**0**	**11**	7.04493	20.8978	27.9427	+	+
**UCHL1**	**0**	**6**	6.30418	21.3505	27.6547	+	+
**LDHB**	**0**	**2**	5.79512	20.3444	26.1395	+	+
**ALDOA**	**0**	**4**	4.30932	21.74	26.0493	+	+
**CLINT1**	**0**	**4**	4.22157	22.6438	26.8654	+	+

Sup. Fig. 4e

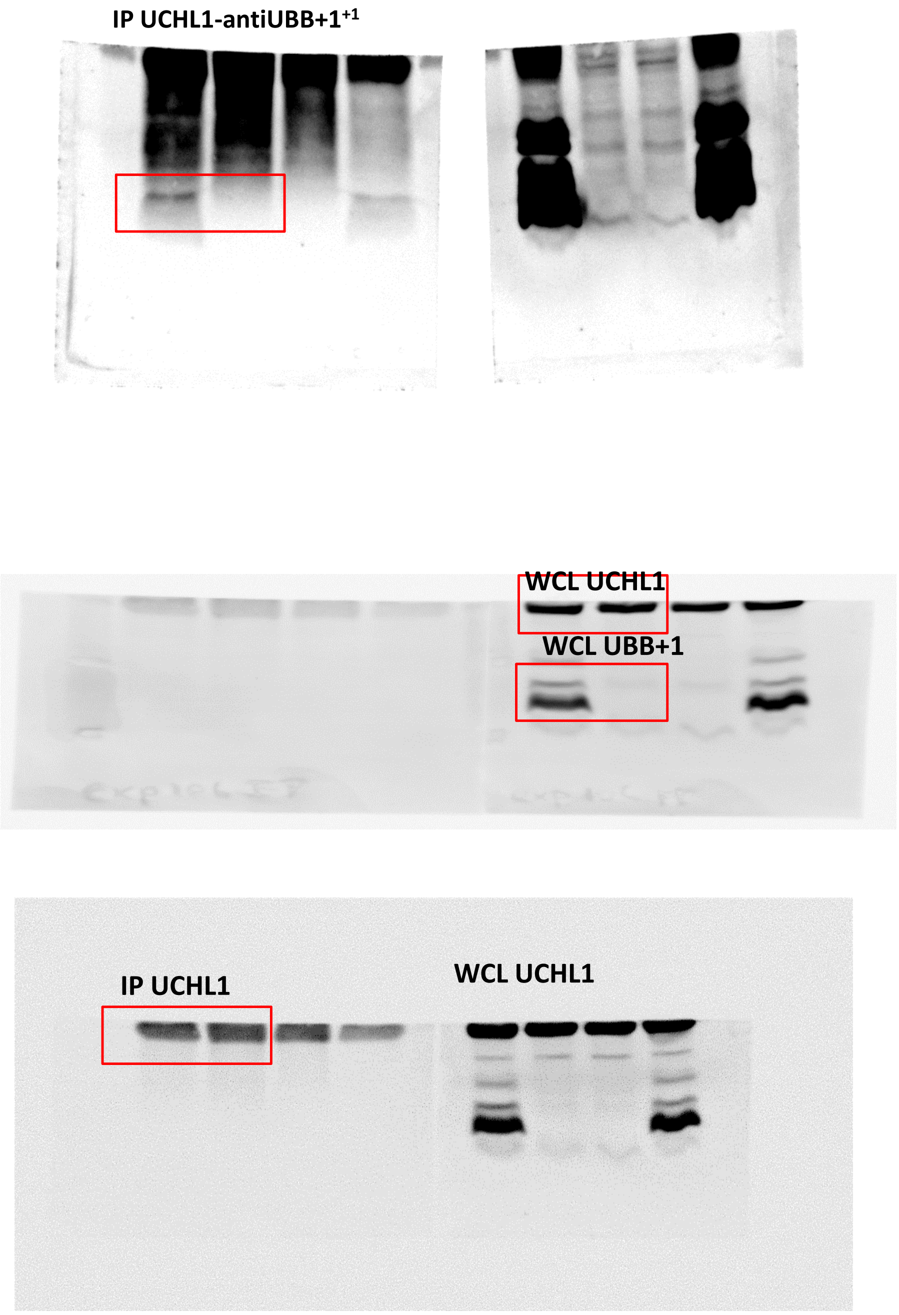

Sup. Fig. 4h

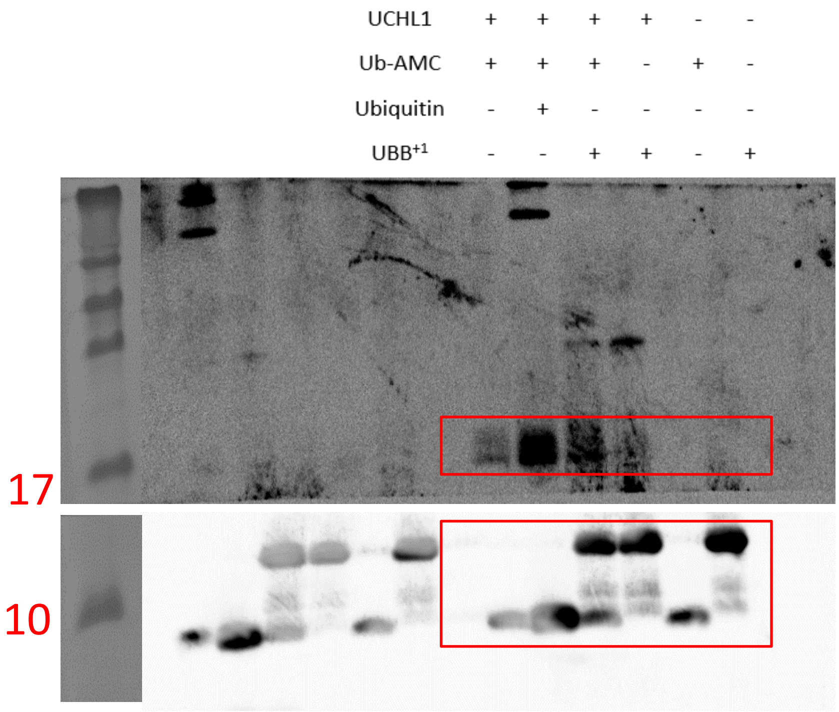

*gel was cut before exposure

Sup. Fig. 4j

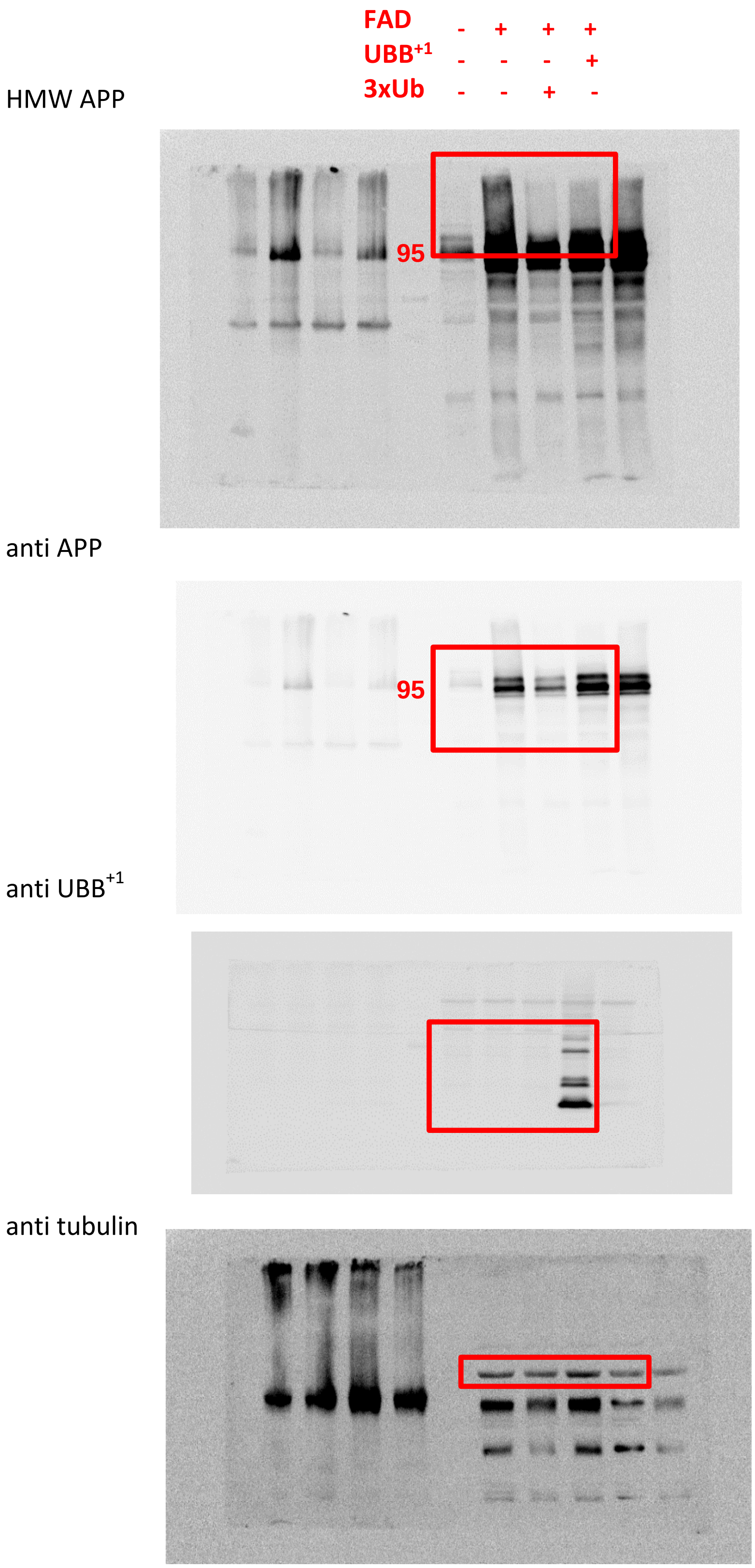

Sup. Fig. 5a

anti GAPDH

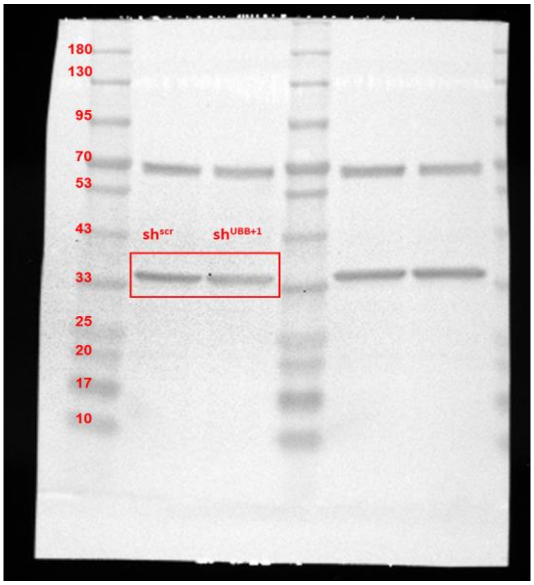

anti UBB^{+1}

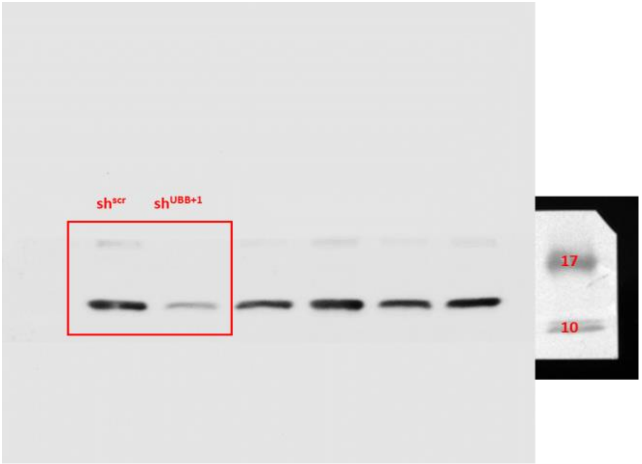

anti ubiquitin

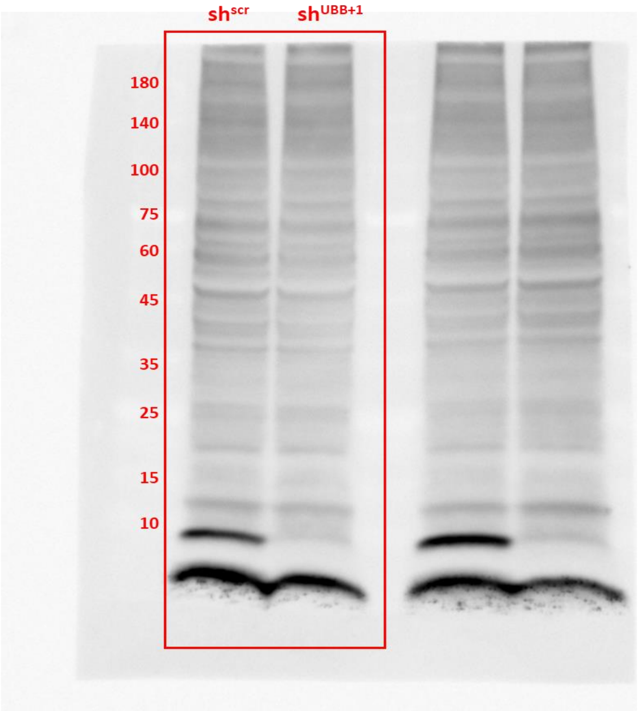

anti GAPDH

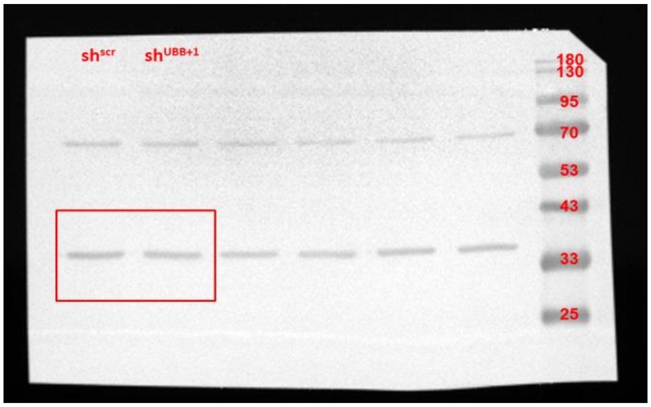

Sup. Fig. 5b

anti Ubiquitin

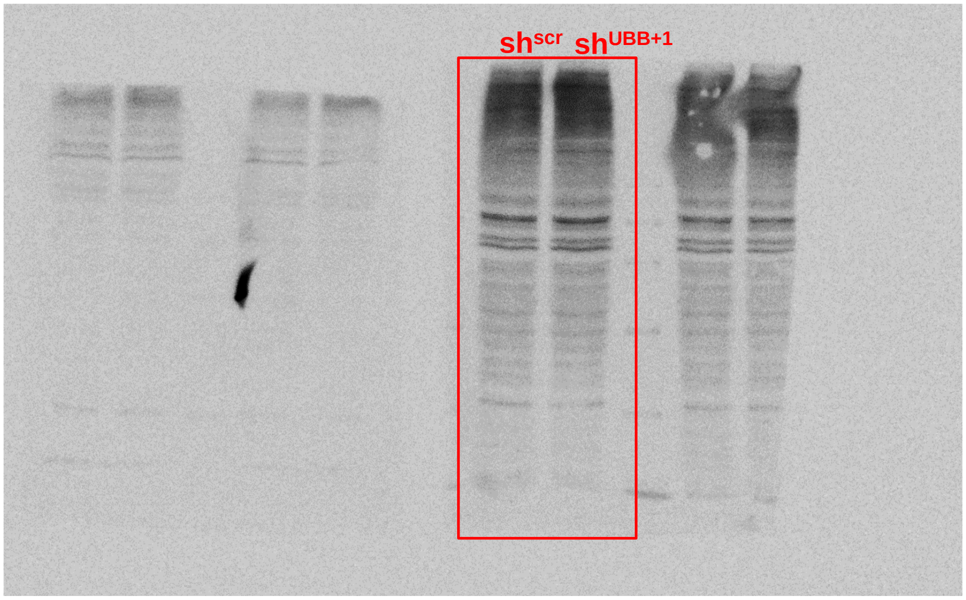

anti Ubiquitin

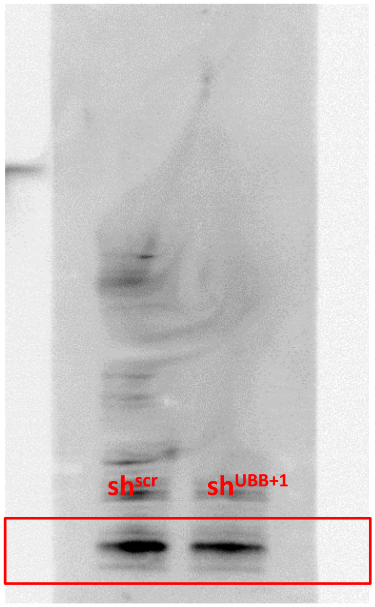

anti GAPDH

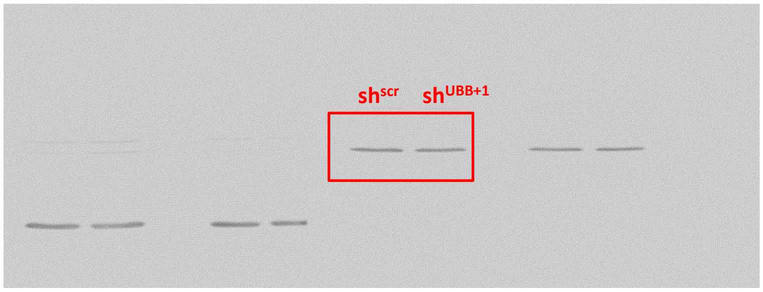

Sup. Fig. 5e

anti UBB+1

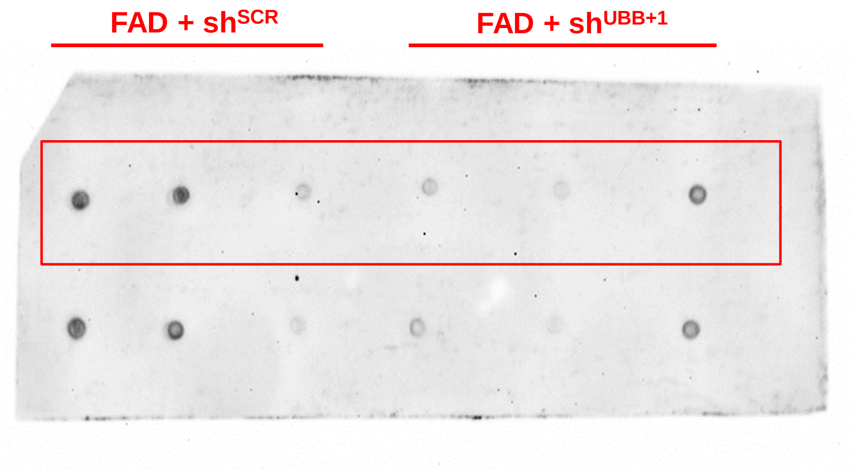

Supplementary fig. 5f

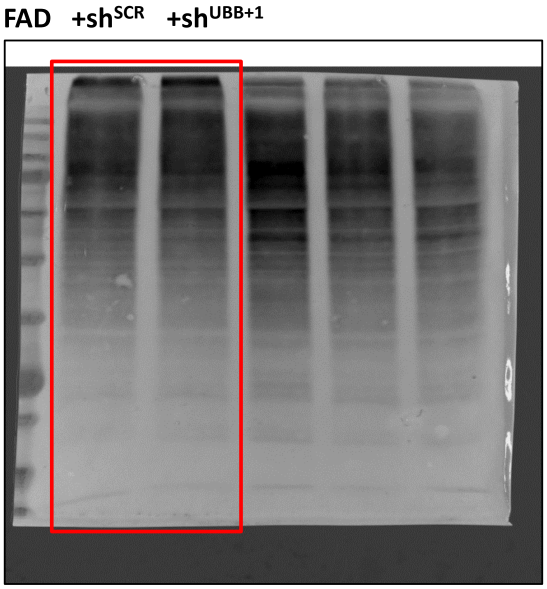

Ubiquitin

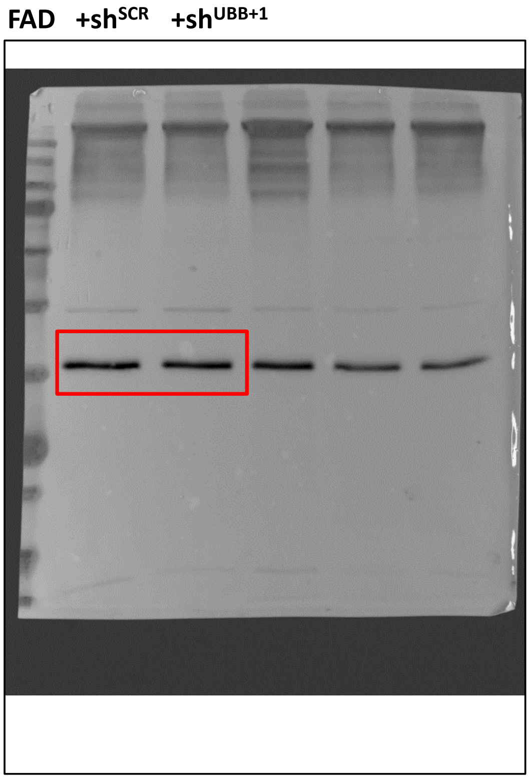

GAPDH

Sup. Fig. 5g

UBB qPCR

	all samples			without scr-3 & sh-1 samples		
Normalized Ubb	Avg normalized	Ratio of Avg	p. value	Avg normalized	Ratio of Avg	p. value
1.420520134						
1.842413264						
1.318128006						
0.279137825	1.215049807	1		1.527020468	1	
2.852384885						
1.549186353						
1.332792308						
1.910210795	1.911143585	1.251550733	0.1907	1.597396485	1.046087147	0.777313316

		Avg	Std	SE	P value
FAD + sh^{SCR}	1.420520134	1.215049807	0.664	0.331958747	0.190745471
	1.842413264				
	1.318128006				
	0.279137825				
FAD + sh^{UBB+1}	2.852384885	1.911143585	0.671	0.335588946	
	1.549186353				
	1.332792308				
	1.910210795				

Sup. Fig. 5i

cCasp3+ particles

	Count	Total Area	Average	% Area	Mean	Avg	p. value
shSCR_cCasp3_1.JPG	556	8358	15.032	2.618	255		
shSCR_cCasp3_3.JPG	474	8188	17.274	2.611	255		
shSCR_cCasp3_4.JPG	454	7111	15.663	2.243	255		
shSCR_cCasp3_2.JPG	497	10157	20.437	3.311	255	495.3	
shUBB_cCasp3_1.JPG	544	9503	17.469	3.063	255		
shUBB_cCasp3_2.JPG	491	7088	14.436	2.233	255		
shUBB_cCasp3_3.JPG	594	10426	17.552	3.256	255		
shUBB_cCasp3_4.JPG	534	10188	19.079	3.308	255	540.8	0.187205

		**Avg**	**Std**	**SE**	**P value**
FAD + shSCR	556	495.25	44.147	22.07	0.1872
	474				
	454				
	497				
FAD + shUBB+1	544	540.75	42.296	21.15	
	491				
	594				
	534				

Sup. Fig. 5j

anti Aβ (6E10)

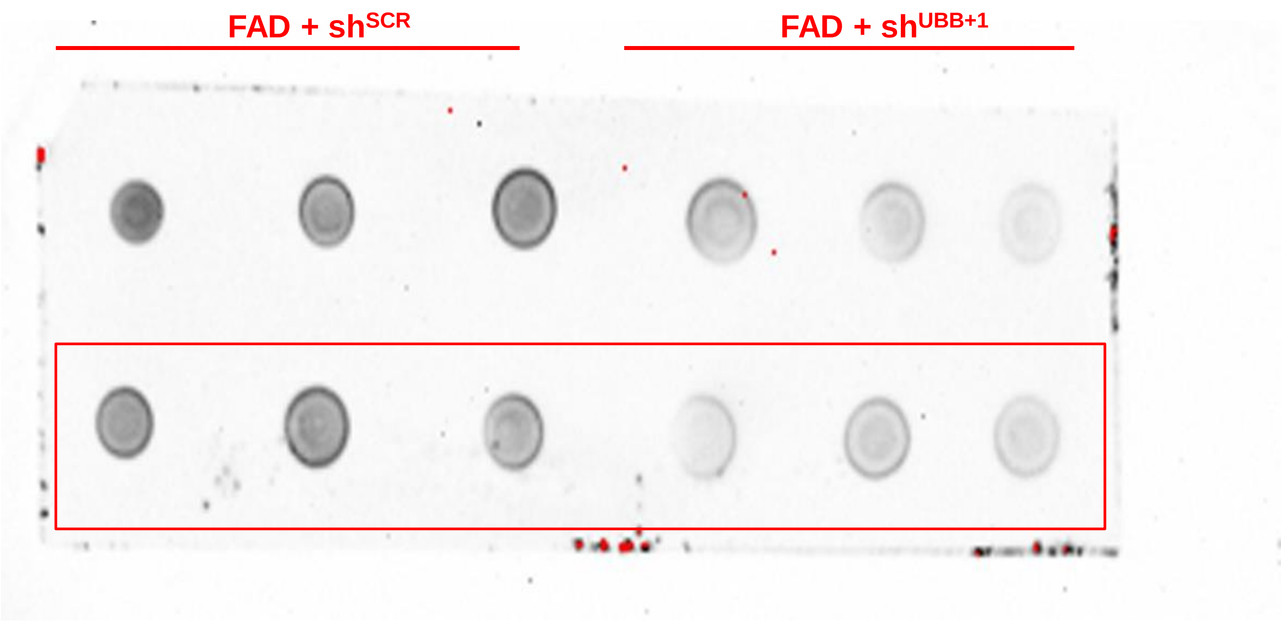

anti β-tubulin III

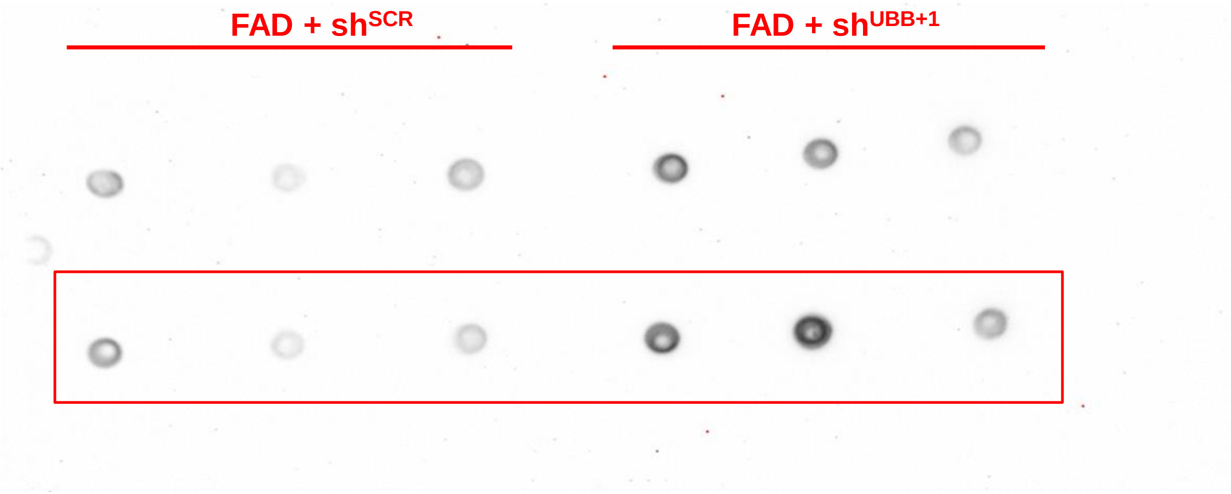

Sup. Fig. 5k

anti p-tau (PHF)

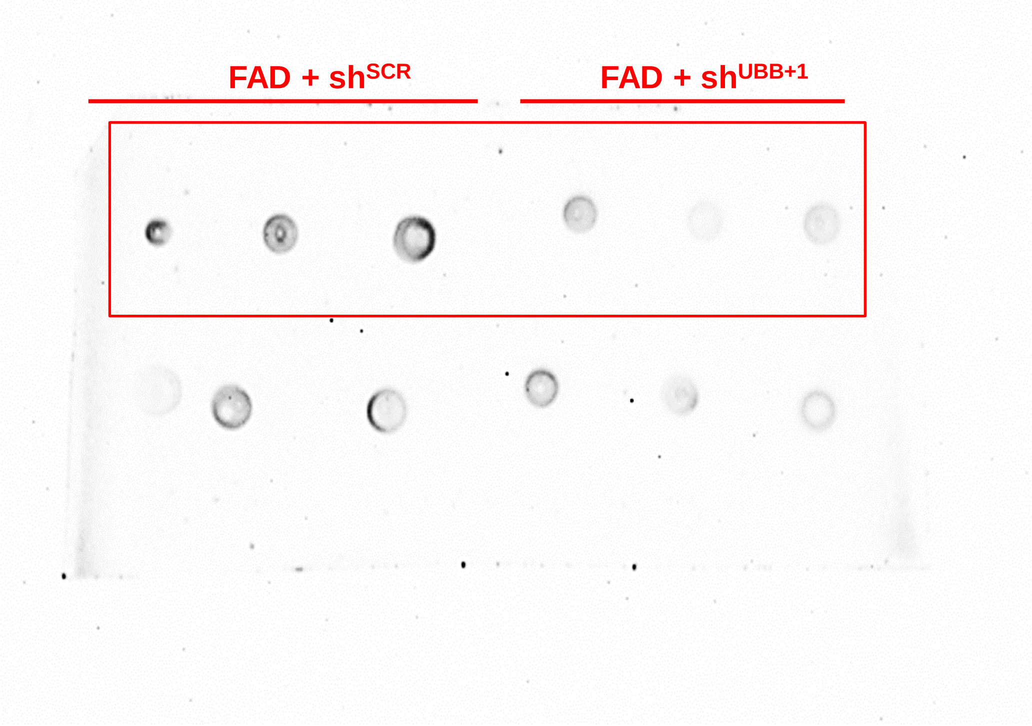

anti total tau (MC1)

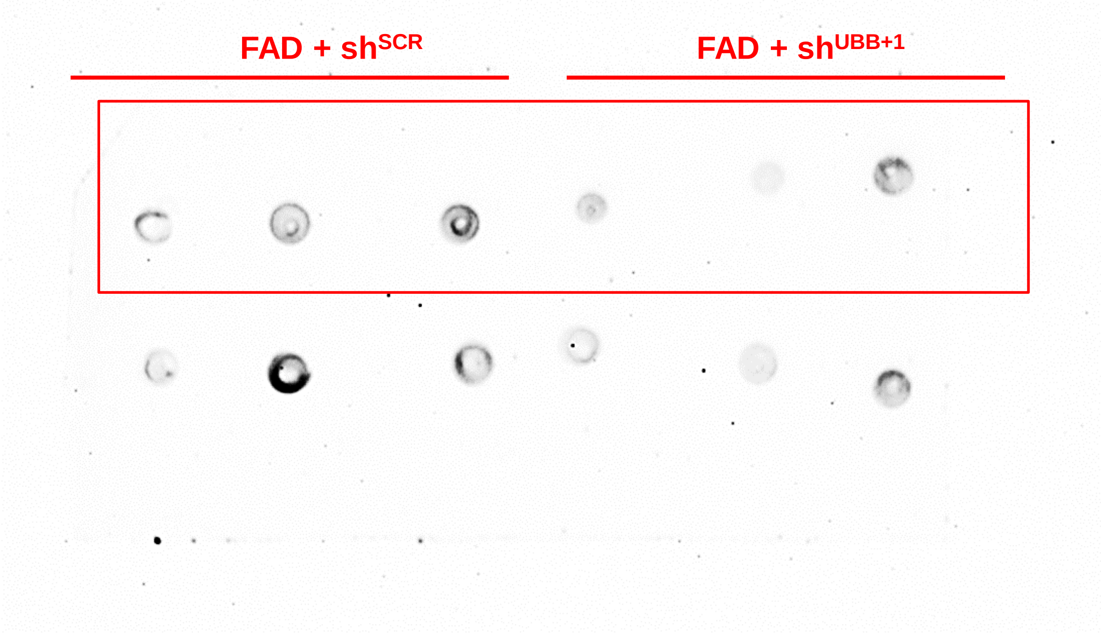

Supplement: Supplementary file 1 — Supplementary Information [file 41467_2023_41545_MOESM1_ESM.pdf]
