# Supplementary material for: Altered ubiquitin signaling induces Alzheimer’s disease-like hallmarks in a three-dimensional human neural cell culture model
Source: Nat Commun. 2023 Sep 22;14:5922. doi: 10.1038/s41467-023-41545-7 (PMC10516951; doi:10.1038/s41467-023-41545-7)
Supplement: Supplementary file 3 — Reporting Summary [file 41467_2023_41545_MOESM3_ESM.pdf]

nature portfolio

Corresponding author(s): Fuchs Y and Glickman M

Last updated by author(s): 09.08.2023

Reporting Summary

Nature Portfolio wishes to improve the reproducibility of the work that we publish. This form provides structure for consistency and transparency in reporting. For further information on Nature Portfolio policies, see our Editorial Policies and the Editorial Policy Checklist.

Statistics

For all statistical analyses, confirm that the following items are present in the figure legend, table legend, main text, or Methods section.

n/a	Confirmed	
☐	☒	The exact sample size (*n*) for each experimental group/condition, given as a discrete number and unit of measurement
☐	☒	A statement on whether measurements were taken from distinct samples or whether the same sample was measured repeatedly
☐	☒	The statistical test(s) used AND whether they are one- or two-sided<br>*Only common tests should be described solely by name; describe more complex techniques in the Methods section.*
☒	☐	A description of all covariates tested
☐	☒	A description of any assumptions or corrections, such as tests of normality and adjustment for multiple comparisons
☐	☒	A full description of the statistical parameters including central tendency (e.g. means) or other basic estimates (e.g. regression coefficient) AND variation (e.g. standard deviation) or associated estimates of uncertainty (e.g. confidence intervals)
☒	☐	For null hypothesis testing, the test statistic (e.g. *F*, *t*, *r*) with confidence intervals, effect sizes, degrees of freedom and *P* value noted<br>*Give P values as exact values whenever suitable.*
☒	☐	For Bayesian analysis, information on the choice of priors and Markov chain Monte Carlo settings
☒	☐	For hierarchical and complex designs, identification of the appropriate level for tests and full reporting of outcomes
☒	☐	Estimates of effect sizes (e.g. Cohen's *d*, Pearson's *r*), indicating how they were calculated

Our web collection on statistics for biologists contains articles on many of the points above.

Software and code

Policy information about availability of computer code

Data collection	Zen lite, ImageQuant", Mascot and Sequest search engine
Data analysis	Image Studio" Lite , Discoverer software version 1.4Z, Zen lite, Prism-GraphPad, ImageJ

For manuscripts utilizing custom algorithms or software that are central to the research but not yet described in published literature, software must be made available to editors and reviewers. We strongly encourage code deposition in a community repository (e.g. GitHub). See the Nature Portfolio guidelines for submitting code & software for further information.

Data

Policy information about availability of data

All manuscripts must include a data availability statement. This statement should provide the following information, where applicable:

- Accession codes, unique identifiers, or web links for publicly available datasets
- A description of any restrictions on data availability
- For clinical datasets or third party data, please ensure that the statement adheres to our policy

This study does not contain data sets.
All other data are available in the main text or the supplementary materials, all figures are accompanied by a source data file.
There are no restrictions on data availability.

Research involving human participants, their data, or biological material

Policy information about studies with human participants or human data. See also policy information about sex, gender (identity/presentation), and sexual orientation and race, ethnicity and racism.

Reporting on sex and gender	Biological material from human participants are described in supplementary table 1.
Reporting on race, ethnicity, or other socially relevant groupings	Biological material from human participants are described in supplementary table 1.
Population characteristics	Biological material from human participants are described in supplementary table 1.
Recruitment	The samples were obtained from an academic depository.
Ethics oversight	MST Hospital Group in Enschede, The Netherlands

Note that full information on the approval of the study protocol must also be provided in the manuscript.

Field-specific reporting

Please select the one below that is the best fit for your research. If you are not sure, read the appropriate sections before making your selection.

- [x] Life sciences
- [ ] Behavioural & social sciences
- [ ] Ecological, evolutionary & environmental sciences

For a reference copy of the document with all sections, see nature.com/documents/nr-reporting-summary-flat.pdf

Life sciences study design

All studies must disclose on these points even when the disclosure is negative.

Sample size	A minimum of three independent replicates were analyzed in the current study providing reproducibility and manageability.
Data exclusions	One sample out of 6 was excluded in APOE mouse lysate due to abnormality
Replication	All the experiments were reliably reproduced as specified in figure legends.
Randomization	Experiments were not randomized. All protocols for tissue culture, immunostaining, and Western blotting were repeated by different scientists in two teams over the course of several years.
Blinding	Investigator was blinded for data analyses of Abeta plaques and p-tau quantification from microscopy images.

Reporting for specific materials, systems and methods

We require information from authors about some types of materials, experimental systems and methods used in many studies. Here, indicate whether each material, system or method listed is relevant to your study. If you are not sure if a list item applies to your research, read the appropriate section before selecting a response.

Materials & experimental systems

n/a	Involved in the study	
☐	☒	Antibodies
☐	☒	Eukaryotic cell lines
☐	☐	Palaeontology and archaeology
☐	☒	Animals and other organisms
☒	☐	Clinical data
☒	☐	Dual use research of concern
☒	☐	Plants

Methods

n/a	Involved in the study	
☒	☐	ChIP-seq
☐	☒	Flow cytometry
☒	☐	MRI-based neuroimaging

Antibodies

Antibodies used	6E10 anti-amyloid-β (Biolegend, Cat. no. 803015)<br>3D6 anti-amyloid-β (Creative Biolabs, Cat. no. PABL-011),<br>GFAP (DAKO, Cat. no. Z0334)<br>Tyrosine hydroxylase (Cell Signaling Technology, Cat. no. 2792)<br>NR2B (Antibodies Incorporated, Cat. no. N/59/36).

UBB+1 (custom-made by Sigma Aldrich for our lab using the C-terminus 20aa sequence)
MAP2 (Abcam, Cat. no ab5392)
UCH-L1 (Abcam, Cat. no. ab8189)
AT8 anti p-tau (Thermo Scientific, Cat. no. MN1020)
C-terminus APP (MERK, Cat. no. 751-770)
Ubiquitin (Dako, Cat. no. Z0458)
PSEN1 (Cell Signaling Technology, Cat. no. 5643)
β-tubulin III (Abcam, Cat. no. AB24629);
GAPDH (Sigma, Cat. no. G9545)
β-actin (Santa Cruz, Cat. no. sc-47778)
MC1 and PHF-1 (kindly donated by P. Davies)
AlexaFluor 488/546 anti-mouse, and -rabbit secondary antibodies (Life Technologies, Cat. no. A-11011, A-11003, A-11034, A-11035).

Validation

All commercial antibodies were validated with a positive and negative control in all experiments. For UBB+1 antibody, we used the same antigen sequence as published (ref #8) and validated by over-expressing UBB+1 in HEK293T cells vs. non-transfected, as well as on recombinant purified UBB+1.

Eukaryotic cell lines

Policy information about cell lines and Sex and Gender in Research

Cell line source(s)

HEK293FT and SK-N-SH cell lines were purchased from the ATCC, RenCells VM neural progenitor cell line purchased from EMD Millipore (SCC008).

Authentication

RenCells VM neural progenitor cell line was validated by Millipore and by our immustaining of neuronal markers. HEK293FT and SK-N-SH cell lines were validated by ATCC.

Mycoplasma contamination

Cell lines have been tested regularly for mycoplasma and are negative

Commonly misidentified lines (See ICLAC register)

No commonly misidentified lines are used in this study

Palaeontology and Archaeology

Specimen provenance

Provide provenance information for specimens and describe permits that were obtained for the work (including the name of the issuing authority, the date of issue, and any identifying information). Permits should encompass collection and, where applicable, export.

Specimen deposition

Indicate where the specimens have been deposited to permit free access by other researchers.

Dating methods

If new dates are provided, describe how they were obtained (e.g. collection, storage, sample pretreatment and measurement), where they were obtained (i.e. lab name), the calibration program and the protocol for quality assurance OR state that no new dates are provided.

☐ Tick this box to confirm that the raw and calibrated dates are available in the paper or in Supplementary Information.

Ethics oversight

Identify the organization(s) that approved or provided guidance on the study protocol, OR state that no ethical approval or guidance was required and explain why not.

Note that full information on the approval of the study protocol must also be provided in the manuscript.

Animals and other research organisms

Policy information about studies involving animals; ARRIVE guidelines recommended for reporting animal research, and Sex and Gender in Research

Laboratory animals

ApoE-TR mice

Wild animals

No wild animals were used

Reporting on sex

male

Field-collected samples

No field collected samples were used

Ethics oversight

Experiments were approved by the Tel Aviv University Animal Care Committee

Note that full information on the approval of the study protocol must also be provided in the manuscript.

Flow Cytometry

Plots

Confirm that:

- [x] The axis labels state the marker and fluorochrome used (e.g. CD4-FITC).
- [x] The axis scales are clearly visible. Include numbers along axes only for bottom left plot of group (a 'group' is an analysis of identical markers).
- [x] All plots are contour plots with outliers or pseudocolor plots.
- [x] A numerical value for number of cells or percentage (with statistics) is provided.

Methodology

Sample preparation	ReN cells grown for 2 passages, dissociated by Accutase (A11105-01, Life Technologies) and centrifuged at 500 × g for 2.5 min. Cell pellets were resuspended in ice-cold PBS with 2% KnockOut Serum Replacement solution (10828028, Life Technologies) and 2% B27 and passed through a cell strainer (352350, 70 µm Nylon, BD Biosciences) to obtain a uniform single cell suspension. After adjusting cells to a density of 3 × 10^6 cells/ml, cell suspensions were applied to FACS-assisted single cell sorting.
Instrument	FACSAriaIIIu cell sorter (BD)
Software	FSC Express Flow Cytometry Data Analysis
Cell population abundance	Cells were sorted by setting a predefined purity mask (16/32) on top 2% of GFP and mCherry positive cells,<br>as determined by histogram for fluorescence intensity (see below). Purity was examined by fluorescence microscopy post sorting (>95%).
Gating strategy	Unstained cell sample was used to set PMT voltages and gating of FSC-A/SSC-A. Dead cells and debris were gated out according to FSC and SSC properties.<br>Out of this parent population singlets were gated using FSC-W vs. FSC-H. Two histograms were used for GFP and mCherry signals to gate for the top 2% (out of all events)<br>cells positive for both GFP and mCherry. Data is shown as top 2% (pink) out of singlets (blue) on GFP-A/mCherry-A. Dotted black line demarcates this population.

- [x] Tick this box to confirm that a figure exemplifying the gating strategy is provided in the Supplementary Information.
